# Social life cycle assessment for industrial product development: A comprehensive review and analysis

**DOI:** 10.1016/j.heliyon.2023.e22861

**Published:** 2023-11-28

**Authors:** Carmen Mármol, Amanda Martín-Mariscal, Alberto Picardo, Estela Peralta

**Affiliations:** Departamento de Ingeniería del Diseño, Escuela Politécnica Superior, Universidad de Sevilla, Virgen de África 7, E-41011 Sevilla, Spain

**Keywords:** Life cycle assessment, Social life cycle assessment, Social development indicators, Industrial products, Socioeconomic impact, Corporate social responsibility

## Abstract

The sustainable development goals outlined in the 2030 Agenda encompass a range of global challenges aimed at promoting prosperity and security for both current and future generations. Within this context, the Social Life Cycle Assessment emerges as a valuable tool for assessing the socioeconomic impact assessment associated with the life cycle of industrial products. Despite the presence of a methodological framework with a structured process, the implementation of the Social Life Cycle Assessment within the industrial domain is hampered by the lack of precise indicators, context-specific databases, limited case studies, and the dynamic characteristics inherent in social data. This study conducts a comprehensive review of the scientific literature and examines methodologies, databases, and software, focusing specifically on its applicability in the design and development of industrial products. The main objective is to evaluate the current research effort, identify available technical and digital resources for implementation in industrial companies, and outline key areas of work and specific challenges related to the social dimension of life cycle assessment. The results emphasize the priority of improving indicator frameworks within specific impact categories and developing novel metrics to mitigate uncertainty in decision-making processes. Furthermore, it underscores the importance of transitioning to standardized application procedures within industrial product sectors, thus ensuring methodological consistency and improving the reliability of assessments.

## Highlights

• The social dimension of sustainability is understudied for industrial products.
• A comprehensive review of the application of S-LCA in industrial products is carried out.
• The main research and the challenges related to the social dimension of LCA are defined.
• Quantitative social indicators improve comparability and decision making.
• S-LCA is a useful method for the management of the life cycle of industrial products.

## 1 Introduction

The United Nations (UN) developed the action plan 'Transforming our world: the 2030 Agenda for Sustainable Development' at the General Assembly on September 25, 2015, which encompasses 17 integrated goals that combine three dimensions: economic, social, and environmental [1]. The goals not only establish global challenges, but also guide the actions of the private sector, civil society, and philanthropic organizations, shaping global priority flows and policies. A comprehensive analysis of these goals and their descriptors reveals that equity and social justice are recurring themes that connect the different SDGs [2,3]. Specifically, Goal 12, Responsible Production and Consumption, primarily focuses on the efficient management of natural resources. However, within the context of this goal, issues such as forced labour, child exploitation, inequality, or occupational health are interconnected and impact responsible production and consumption practices worldwide [4]. Therefore, in recent years, the integration of the SDGs into corporate mission and vision statements has gained traction, suggesting a growing recognition of the need to consider both social and environmental concerns. This highlights the need for a more thorough analysis and control of social impacts, an area still under active development within the sustainable development field [5,6].

According to the goals of the 2030 Agenda, regulating the social and socioeconomic impacts of consumer goods is relevant to achieve a fair society. Product development, as a process of creating new goods or services or improving existing ones, involves the management of the entire life cycle, including decision making related to material extraction, production, logistics, use, and end of life. The product life cycle can be a significant source of environmental, social, and economic impact. Sustainable product development projects that focus on efficient resource use, social responsibility, and user well-being are essential for achieving the SDGs [7]. In the near future, companies should comply with social requirements in product design, necessitating governments regulations and the development of adequate assessment tools. Currently, the framework for the development of socially sustainable products is optional. While the ISO 26000 standard on corporate social responsibility is available at the organizational level, there are no regulations applicable to the product life cycle, the existing design and evaluation methods are limited and there are no established certification ecolabels that guide socially responsible consumer choices. In addition, there is a gap between the combinations of methods for the analysis of environmental, social, and economic impact (Life Cycle Sustainability Assessment - LCSA) and the evaluation of the Sustainable Development Goals (SDG) [8]. This situation makes it difficult for manufacturing and distribution companies to notify users of the benefits of their product portfolios, and consequently to promote social responsibility in consumption.

The first life-cycle assessment with an environmental approach (LCA) was carried out in the early 1970s, but standardization efforts began in 1990 by the SETAC (Society of Environmental Toxicology and Chemistry). In 2003, UNEP and SETAC recognized the need to integrate social criteria into LCA. Social LCA (S-LCA) approaches were actively explored and in 2009 the first guidelines on the methodological approach were developed. When analyzing previous methods, the main and most widely used is the Social Life Cycle Assessment [[9], [10], [11]].

Although guidelines such as the 'Guide for the Social Life Cycle Assessment of Products' [12,13] are available, the implementation of the S-LCA within the industrial domain is hampered by the lack of precise indicators, context-specific databases, limited case studies, and the dynamic characteristics inherent in social data. Analysis indicators are numerous, involving sequential tasks that require a substantial time investment, based mostly on information from the product life cycle [14] and relying on conceptual and qualitative data. Qualitative results are difficult to interpret and are generally associated with high uncertainty. These drawbacks make their use in engineering problem solving complex, where results must be measurable, precise, and comparable. A thorough literature review in S-LCA can provide insights into overcoming these limitations, enabling the development of more quantitative and structured approaches in future research.

To address the need for a particular and quantitative process for applying S-LCA in industrial projects, this paper conducts a comprehensive review of the scientific literature and examines available tools, methodologies, databases, and software related to LCA. The aim is to explore the state-of-the-art of the social life cycle analysis methodology applied to the design and development of industrial products to: 1) understand the current research effort; 2) identify the availability of technical and digital resources for its application in companies and industrial organizations; and 3) define the main challenges related to the social dimension of LCA. Despite its significance, scientific literature on applications of S-LCA in the industrial sector remains limited. While there are studies exploring this approach in other contexts [[15], [16], [17], [18], [19], [20]], cases illustrating the implementation of S-LCA in industrial products are scarce [[21], [22], [23], [24], [25], [26], [27], [28]]. Previous review works [14,[29], [30], [31], [32], [33]] have delved into S-LCA extensively, yet none have taken a specific approach to the product lifecycle, exploring the latest updates available in this field. Therefore, this study identifies recent research fields related to Social Life Cycle Assessment and Sustainable Development Goals. It categorizes and analyzes various methodologies and approaches used to measure social impacts in the life cycle of industrial products, including the latest updates focusing on new impact categories proposed in recent guidelines. The study also examines the implications and challenges associated with integrating these categories into S-LCA. Among these challenges, technological and digital resources play a fundamental role in product design, analysis, and development. Assessing the availability and usability of these resources bridges the gap between theoretical knowledge and practical application. In this context, this work is valuable as it explores how S-LCA methods can be integrated into organizations and industrial processes. Understanding these challenges is essential to provide a roadmap for future research efforts and industrial applications, creating specific strategies that ensure research aligns with the industry's most pressing needs.

The article is structured as follows: Section 2 describes the review methods; Section 3 presents the results structured according to the scope of the research, including the foundations and fundamental principles, methodologies, standards, indicators, calculations, and case studies. Finally, Section 4 discusses the results, identifying research effort and future work lines, and Section 5 sets out the main conclusions of the study.

## 2 Methods

**Fig. 1:**
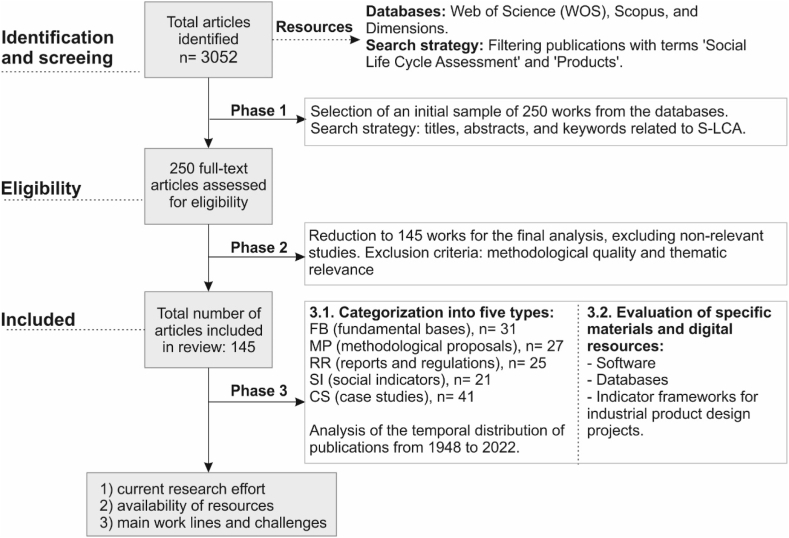
Review methodology.

Fig. 1 presents the complete procedure in three phases followed in the review of S-LCA applied to industrial products.

**Fig. 2:**
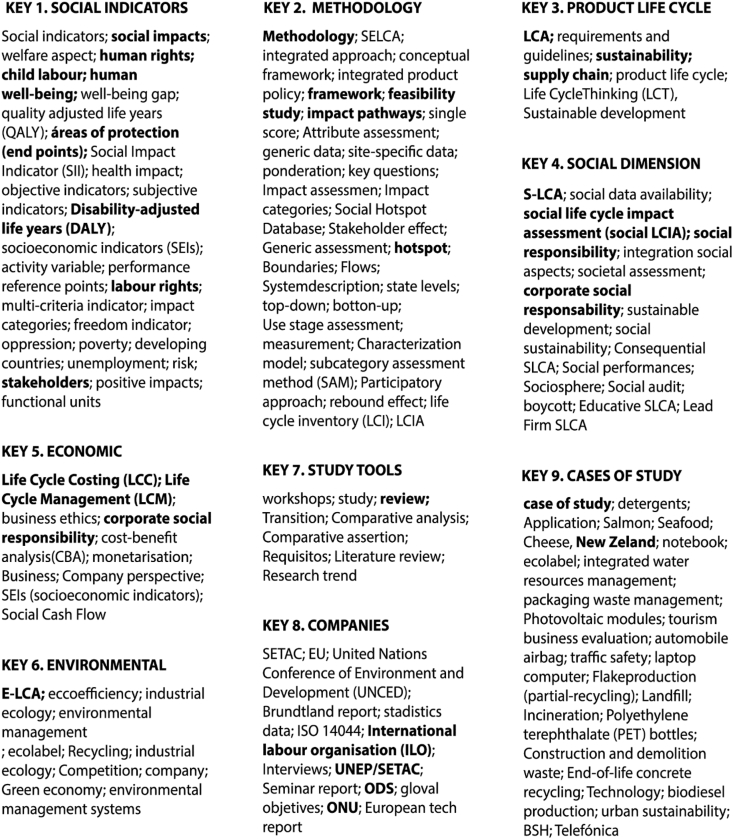
Keywords thesaurus in the sample studied.

The primary sources used were Web of Science (WOS) databases, Scopus, and Dimensions databases. The search strategy focused on titles, abstracts, conclusions, and keywords related to S-LCA, particularly its application to the life cycle of industrial products (see Fig. 2). Data collection, selection, analysis, and visualization were carried out using 'Microsoft Excel' and 'VOSviewer'. In the initial stage (identification and screening), Dimensions.ai software was used to search for publications with the terms 'Social Life Cycle Assessment' y 'Products'. This search was narrowed down to 3052 items published from 1990 (approximately the beginning of LCA methodology in the scientific network) until the end of 2022. The publication dates for this initial analysis were not limited to obtain a comprehensive historical evolution of the methodology.

Of the total articles found, in phase 1 (see Fig. 1, eligibility) an initial sample of 250 work was selected from the WOS and Scopus databases and then analyzed. Finally, in phase 2 (see Fig. 1, “included”) 145 works were chosen for inclusion in the study. Exclusion criteria included the methodological quality of the studies and thematic relevance (excluding publications that were not directly related to the Social Life Cycle Assessment of industrial products or services). Subsequently, in phase 3 a critical review of the sample was carried out, classifying the articles according to their relevance and contribution to the S-LCA for industrial products. It should be noted that additional supporting documents, including guides, standards, and regulations relating to S-LCA methodology, were added to the final sample. Five relevant groups were identified for S-LCA, which served as the basis for the classification and analysis of the final sample.

• Fundamental bases (FB) with articles that establish the foundations and principles of the S-LCA. 31 articles were selected in this group, providing conceptual support for the study. It includes state-of-the-art and S-LCA reviews, studies of life cycle thinking (LCT), and complementary work on life cycle assessment in the environmental (LCA) and economic (LCC) dimensions.
• Methodological proposals (MP) with works that analyze, develop, or propose new S-LCA procedures. A total of 27 articles were selected.
• Reports and Regulations (RR): This group includes various reports and declarations of international organizations on social issues of interest to the S-LCA. It also includes regulations on environmental LCA and various original guidance documents. 25 documents were collected.
• Social indicators (SI): This group includes articles that focus on different indicator frameworks, proposals for impact indicators, and the state-of-the-art of existing indicators. These works are essential for understanding the current state of frameworks available for calculating social impact in industrial product design projects. 21 articles were extracted in this category.
• Case study (CS): These studies provide a practical foundation for the S-LCA methodology. Additionally, this group was analyzed to find out which industrial sectors apply S-LCA. A total of 41 articles completes this group.

Finally, the state-of-the-art of S-LCA was completed based on the analysis of existing electronic materials, including resources such as application software and databases, as well as other supporting elements, such as guides and regulations. Special emphasis was placed on examining the social indicator frameworks focused on implementing an S-LCA in industrial product design projects, as it is one of the main objectives of this study.

The results are presented in section 3 with the following structure: bibliometric analysis, and assessment of (I) fundamentals and principles of S- LCA; (II) S-LCA methodologies; (III) normative and standardization; (IV) socioeconomic impact categories and indicators; (V) resources and development tools, including calculation tools, software, and databases; and (VI) case studies.

## 3 Results

### 3.1 Bibliometric analysis

**Fig. 3:**
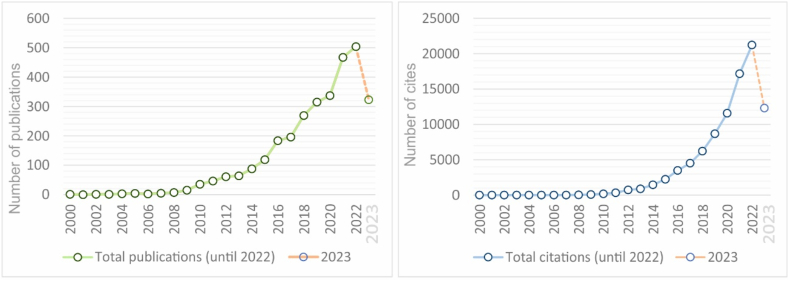
Number of publications (left) and citations (right) of 'S-LCA' and 'products'.

The first bibliometric analysis leading to the following findings. S-LCA is a hot topic with a significant increase in research effort since 2016. The number of articles published per year and citations has shown steady growth, as summarized in Fig. 3.

**Fig. 4:**
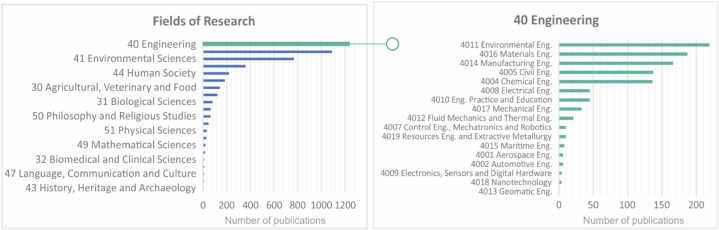
Number of publications on “S-LCA” and “products” classified by research field.

The bibliographical analysis (Fig. 3, Fig. 4) reveals a significant landscape for S-LCA. The domain of 'Engineering (40)' leads other areas with a total of 1236 publications, highlighting its importance within research programs. This is due to the interest in understanding the social aspects associated with engineering and the utility of assessing social impacts stemming from technological advancements. Within this field, 'Environmental Engineering (4011),' followed by 'Materials Engineering (4016)' and 'Manufacturing Engineering (4014),' are emerging areas with 219, 187, and 166 publications, respectively. These subfields have a direct impact on industrial efficiency, labor conditions, and the local and global economy.

**Fig. 5:**
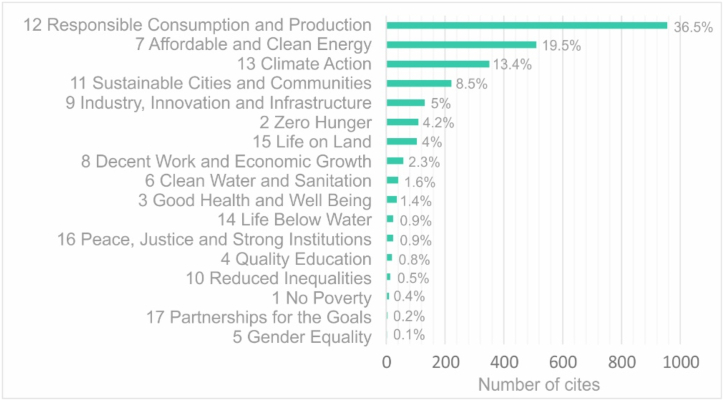
Number of publications on “S-LCA” and “products” by SDG.

When analyzing the scope of each publication in relation to the SDGs, most publications align with SDG 12 'Responsible Consumption and Production', with 516 articles, followed by SDG 7 'Affordable and Clean Energy', with 447 articles, SDG 13 'Climate Action' and SDG 11 'Sustainable Cities and Communities', with 289 and 223 articles, respectively. These results are shown in Fig. 5.

Table 1 presents the top ten most frequently published journals in the field of S-LCA. *Journal of Cleaner Production* leads with 239 publications, followed by Sustainability with 239 articles. *The International Journal of Life Cycle Assessment* also stands out for its variety of publications related to S-LCA.

**Table 1:** Main journals dedicated to the field of study of the S-LCA.

Position	Source titles related to search	Publicacions (total 3052)
1	Journal of Cleaner Production	12 %
2	Sustainability	9 %
3	The International Journal of Life Cycle Assessment	9 %
4	Journal of Industrial Ecology	3 %
5	Sustainable Production and Consumption	2 %
6	Procedia CIRP	2 %
7	The Science of The Total Environment	2 %
8	Resources Conservation and Recycling	2 %
9	Renewable and Sustainable Energy Reviews	2 %
10	Energies	1 %

**Fig. 6:**
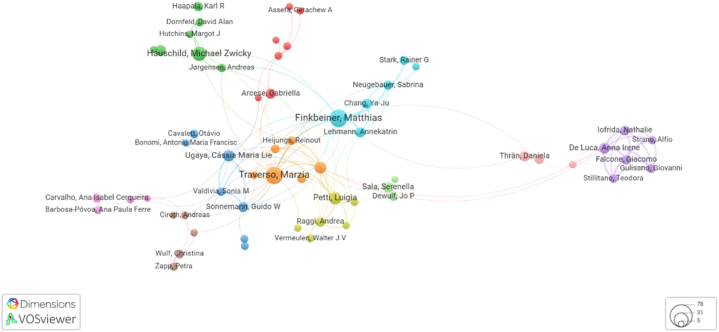
Co-authorship analysis for ‘S-LCA’ and ‘products’ limited to 100 authors.

To understand the scientific trajectory in S-LCA, a co-authorship analysis was conducted using VOSviewer software. Fig. 6 presents a visual map of the most published authors and their relationships. Matthias Finkbeiner from the Technical University of Berlin is the most prominent author with 48 articles on S-LCA. Furthermore, the analysis identified 10 groups of authors with a high level of commonality, distinguished by the different colours on the map.

**Fig. 7:**
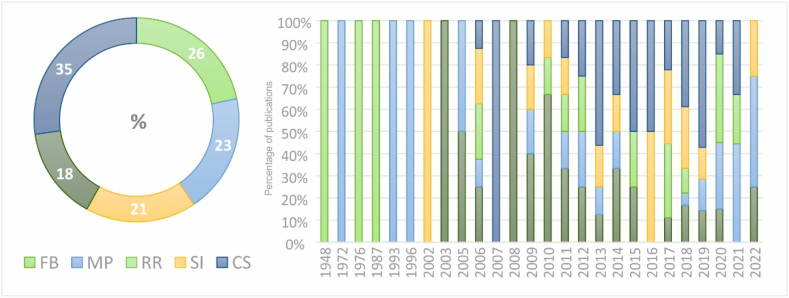
Number of articles in the sample according to scope (left) and distribution by year (right).

Finally, the 145 articles were classified into five categories based on their scope: articles referring to fundamental bases (FB), methodological proposal (MP), standard and reports (RR), social indicator (SI), and case studies (CS). The results indicate that 26 % of the collected references belong to the category FB, 23 % to MP, 21 % to RR, 18 % to SI and 35 % to CS. Fig. 7 shows the classification of the articles in the sample according to their typology and the frequency of publication over the years. The time span is wider than in the first sample, starting from 1948, as it includes documents referred to significant reference resource. In addition, it is noted that most of the articles collected are published during the year 2020. Furthermore, it is worth mentioning that since 2006, there has been a noticeable increase in research interest in S-LCA, evident in a well-balanced exploration of studies encompassing various scopes (FB, MP, RR, SI, and CS).

### 3.2 Basis and fundamental principles of S-LCA

Articles classified as fundamental bases (FB) were analyzed according to scope, including: (1) Social LCA (55 %); (2) Environmental LCA (10 %); (3) Life Cycle Cost, LCC (3 %); and (4) other works which comprise feasibility studies of the methodology and combination of different approaches.

In this field, it is worth highlighting the work of Toniolo et al. [34] on Life Cycle Thinking (LCT), Social Life Cycle Analysis (S-LCA), Environmentally Focused Life Cycle Analysis (LCA or ELCA) and Life Cycle Cost Analysis (LCC) tools. The study clarifies their origins in detail, specifies their advantages and disadvantages, and outlines future challenges and research directions for the scientific community. Worth noting is the study by Finkbeiner et al. [35], where a state of the art on methodologies is elaborated. The paper concludes that a consistent and robust framework of social indicators for S-LCA and LCC is necessary, one that is in balance with existing knowledge on the environmental approach of LCA. The authors also identify the complexity of quantifying social issues in each individual social field (human rights, working conditions, health and safety, cultural heritage, governance, socioeconomic repercussions). This is because the available data indicators are aggregated; therefore, they do not provide a comprehensive representation of each social issue (e.g., Human Development Index (HDI) covers educational levels, health levels, and living standards). Currently, this framework remains incomplete and undeveloped [6]. On the other hand, Li, Wang and Yan [36] conducted a bibliometric keyword analysis of 2637 articles in the field of bioenergy to locate social hotspots. Finally, the study of Huarachi et al. stands out [37], analyzing the historical evolution of the S-LCA in four phases. The first three stages are marked by the publication of specific documents such as 'Social and environmental life cycle assessment' [38], the first UNEP-SETAC guidelines [12], and the methodological sheets [39], respectively. They conclude that the method harmonization is needed. This standardization is justified by the increase in S-LCA publications since 2017, as well as the application of different S-LCA approaches or databases. Trends are analyzed through the evolution of the most used keywords, anticipating an increased use of the SHDB (Social Hotspots Database) and PSILCA (Product Social Impact Life Cycle Assessment database). Currently, focus future research efforts must focus on the development of the calculation process of social impacts in the product life cycle, improving the existing frameworks for impact assessment, such as the Subcategory Assessment Method (SAM).

### 3.3 Analysis of methodologies for S-LCA

In addition to the documents that collect the fundamental bases on S-LCA, there are numerous articles in the scientific literature that propose new approaches or models [38,[40], [41], [42], [43], [44]] or adaptations according to different scopes [[45], [46], [47], [48], [49]]. The analysis of this type of wok is intended to gain a better understanding of the evolution of the methodology along its different streams. The status of the methodology and the progress that has been made can be listed below. Reviewing the history of S-LCA, the first methodological proposals were developed in combination with the environmental LCA, to achieve the calculation of integrated socio-economic and environmental impacts; highlight the methodological proposals “Social and environmental life cycle assessment (SELCA)” [38] y “The extended life-cycle assessment” [50]. Currently, the most developed methodology to measure sustainability in the whole life cycle is the Life Cycle Sustainability Assessment (LCSA), which combines environmental, social, and economic LCA [51]. However, focusing on S-LCA, the methodological process most developed and published is the one proposed by the UNEP-SETAC Guidelines. These differentiate two ways of calculating the LCA according to the objective and scope of the study: (1) S-LCA type I (or reference scale) and (2) S-LCA type II (or impact pathway S-LCA).

- Benchmark scale approach (Type I S-LCA): It proposes an assessment of the different impact sub-category indicators through a comparison with a scale that is taken as a reference. Each level of the scale is defined through the PRPs (Performance Reference Points) or specific benchmarks of the envisaged activity. It is an immediate measure, that is, it does not delve into long-term effects. It is the most current approach and yet the most widely used in case study applications. As examples, the S-LCA of a solar plant [52], S-LCA of a T-shirt purchased in the Netherlands [53] or the comparison of two biomass power generation systems [54].
- Impact path approach (S-LCA type II): Focusses on predicting the consequences of the product system through the characterisation of potential social impacts, or social risks. This is achieved on the basis of a characterisation model in which the specific cause-effect chain is defined, also called impact pathway. In this way, the midpoints and endpoints of the impact chain can be calculated. This is the oldest approach, coinciding with the one established for the calculation of environmental impacts. It is often used in methodological research and in the proposal of more robust indicators. However, there are also case studies that use it, although they are less common [55]. Examples include the S-LCA of tertiary education as a form of poverty alleviation through its corresponding impact pathway [56] or the proposal of robust composite indicators to estimate the impacts of housing rehabilitation [57].

Currently, the most widely used procedure for the S-LCA methodology is the one proposed in the guidelines updated in 2020 by UNEP-SETAC for the Social Life Cycle Assessment of Products and Organizations [13]. This methodology is structured in the phases of an LCA with an environmental focus [[58], [59], [60]]. The adaptations made in the social approach are summarized below.

- **PHASE 1. Goal and Scope.** This phase includes the purpose of the study and the methodological framework. First, the objective of the study and the target audience are selected. The scope of the study is defined, consisting of a series of decisions to be taken in relation to the following phases of the S-LCA: functional unit, reference flow, system output, activity variable, stakeholder and participation strategy, impact categories/subcategories, and final set of indicators, type of LCIA method, and data collection strategy.
- **PHASE 2. Life Cycle Inventory (LCI) Analysis.** This is a data collection phase within the system boundaries based on the flow diagram for the system-product delineated in phase 1. If a quantitative analysis is required, the amount of material, energy or service flow must be quantified for each unit process. Next, the reference flow is determined, where the quantities of each process and flow are taken to comply with the UF, based on linear relationships. However, if appropriate, it is advisable to collect data on the activity variable. This variable is used to reflect the participation of the activities associated with each unitary process. In this way, the percentage share of each process in the system-product is clarified. It can also be used to report on the relative influence of each social attribute in the final stage (phase 4), where the S-LCA results are reported. For each process, a social inventory data is obtained, related to the stakeholders or interested groups (workers, consumers, local community, society, value chain actors, and children).
- **PHASE 3. Life Cycle Impact Assessment (LCIA).** The objective is to estimate, understand, and assess the magnitude of potential social impacts (current, past, or future) of a product system. These are potential impacts, rather than actual ones because they are based on a number of assumptions that involve some uncertainty. Currently two different approaches in S-LCA can be applied. On the one hand, the benchmarking approach is the most frequently in terms of case studies, although it is the most recent approach. However, the impact pathway strategy focusses on research, although it also presents possible case-studies applications. It should be noted that there are no standardized characterization models (that is, accepted through a common consensus by the scientific community) that relate stakeholders or interested groups (workers, consumers, local community, society, value chain actors and children) and impact categories (human rights, working conditions, health and safety, cultural heritage, governance, socio-economic repercussions); this implies that the resulting S-LCA models are generally not well developed [6].
- **PHASE 4. Interpretation.** The results of previous phases are reviewed according to the objective and scope. ISO 14044 defines the following in relation to the interpretation stage: "analysis phase in which the findings of the inventory analysis or impact assessment, or both, are evaluated in relation to the defined objective and scope, in order to reach conclusions and recommendations" [58].

As an alternative to the UNEP guidelines, S-LCA methodologies have been developed from private initiatives. In the scientific literature, there are proposals for frameworks [44,[61], [62], [63], [64], [65], [66]], methodologies with new indicators and category analyses [[67], [68], [69], [70]], integration of social and environmental analyses [48,62,[71], [72], [73], [74]] and specific methodologies for industrial products [12,13,46,53,[75], [76], [77], [78], [79]]. As a reference, the Handbook for Product Social Impact Assessment (PSIA) [80] and the SEEbalance © method stand out. PSIA is an initiative developed by the association of private companies, due to the absence of standardisation for the measurement of global social impacts at the product level. This manual includes the S-LCA as part of the measurement process combined with the SDGs and the principles proposed in the GRI guidelines (principles of balance, materiality, reliability, among others). The handbook also sets out 12 key principles for companies to socially assess their products. However, no case studies were found in the sample of articles in which this method is applied. Besides, SEEbalance ©: BASF, in cooperation with the Universities of Karlsruhe and Jena as well as the Öko-Institut Ev, developed the SEEbalance © method for Socio-Economic Efficiency Analysis in 2004. This method reinterprets the product-related concepts of eco-efficiency and socio-efficiency. It relates ecological and social impacts, which occur throughout the product life cycle, to the end-customer's costs, 'total costs of ownership' involved in the purchase, use, maintenance, disposal or resale of the product (Schmidt et al., 2004). Similarly, there is a more up-to-date version specific to agricultural products: AgBalance [81]. However, no scientific case studies applying this method were found in the sample of articles.

### 3.4 Analysis of standars and regulation for S-LCA

The International Organisation for Standardisation (ISO) unified the LCA methodology and its [82] application in a series of four successive standards, from ISO 14040 in 1997 to ISO 14044 in 2000. Currently, the reformulations in 2006, ISO 14040 “LCA-Principles and framework” and ISO 14044 “LCA-Requirements and guidelines” coexist as the reference documents for the application of LCA [5,58,83]. As mentioned in Section 3.2., the Social Life Cycle assessment is based on the Environmental Life Cycle Assessment [84]. Therefore, they share the same ISO 14040/44 framework, the only existing standardisation of these methodologies. As a consequence, an equivalent structure is maintained in both methodologies. This scheme consists of a four-phase process applied iteratively until the desired specification and coherence is achieved. Some of these common aspects include handling large amounts of data, using the functional unit to develop the inventory, identifying critical points, and conducting third-party reviews.

Throughout the evolution of the S-LCA, the most referenced document has been the 'Guidelines for Social Life Cycle Analysis' [12]. It provides guidance and key aspects for carrying out a full S-LCA, explaining the completion of each phase of the methodology. Objective and Scope, Life Cycle Inventory, Life Cycle Assessment, and Interpretation of Results. It also suggests a framework of impact sub-categories, related to the stakeholders. The impact on each sub-category is characterized using social indicators (qualitative, quantitative, and/or semi-quantitative); this indicator framework is further developed in the document subsequently launched by the same initiative 'Methodological Sheets' [39].

The success of the guidelines is also due to their permanence in the history of the S-LCA, not until just over ten years later, in 2020, the publication of their update [13]. It includes the advances and new paradigms that have arisen throughout the maturation of the methodology, extending the methodological proposal to an organisational level through 'Social Organizational Life Cycle Assessment' (SOLCA) [42].

However, the methodological sheets are consistent guidelines to support the application of the S-LCA, especially in specific contexts [39,85]. Provides an exhaustive study of each impact subcategory, showing -in addition to a proposal of indicators-the types of data, generic and specific collection sources, and additional documents to go deeper (Table 2). This is the reference document for inventory analysis in the S-LCA. For this reason, it does not cover the LCIA and Interpretation of results phases. Although possible extensions are foreseen considering characterisation and aggregation models. They were conceived as a living resource, intended to be expanded and improved over time. Finally, in December 2021, the update of the methodological sheets was published by the same life cycle initiative.

**Table 2:** Information presented in the 'Methodological Sheets' for each subcategory.

Subcategory	Information
Definition:	Social aspects to be considered, scope of the assessment
Policy relevance:	Relationship to sustainable development
Data evaluation:	Related international conventions (sources of enlargement) both general and specific to the subcategory
Inventory Indicators:	Data Types with examples
Limitations	Data sources include both generic (governmental, intergovernmental, and non-governmental organizations) and site-specific data (gathered through interviews with workers, trade union associations, human resources officials, and company-specific documents such as GRI reports).

### 3.5 Analysis of socio-economic impact categories and indicators

**Fig. 8:**
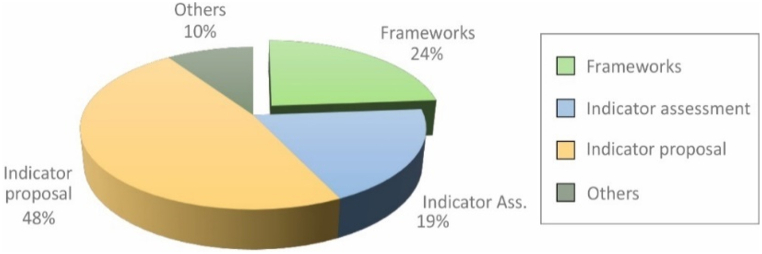
Research effort on impact indicators development.

From bibliographic selection, articles focused on the calculation of social impact were analyzed. This type of publication was given special emphasis, due to the objective of this research, which is to understand the current state of the application of the S-LCA to industrial product development projects. These publications refer to: 1) the state of the art of indicators; 2) proposed frameworks of reference indicators; and 3) development of individual indicators. Fig. 8 illustrates the research effort related to the development of social impact calculation indicators.

Indicator frameworks are the sets of metrics proposed relating to each social impact category. They play a key role in standardizing methodology, as they establish what should be measured so that studies of S-LCA can be comparative. However, the frameworks are currently numerous and optional. In this review, the most holistic ones were considered, that is, those not focused on a particular sector or a specific application. Table 3 show the five general and several specific frameworks selected. Most of the publications on indicators that analyze the fundamental principles of calculating social impact consider as the main reference source the UNEP-SETAC 'Methodological Sheets' [39]. Fürtner et al. highlight that the 33 most commonly used impact categories in the literature fully cover all 31 categories of the UNEP-SETAC framework [68]. However, Kühnen et al. [14] in their study on measuring social performance in product life cycle and supply chains show the highest percentage of indicators based on categories other than the 'Methodological Sheets'. These results are contrasted when analysing the available social LCA indicators, without considering the product life cycle; in this case, the Global Reporting Initiative Sustainability Reporting Guidelines in their G3, G3.5 and G4 versions [86] are the most widely used.

**Table 3:** General frameworks.

Frameworks	References
UNEP&SETAC 2020	[13]
Sustainable Development Goals	[1]
Social Welfare	[87]
SeeBalance	[88]
PSIA	[80]
Others	[45,56,89]

**Fig. 9:**
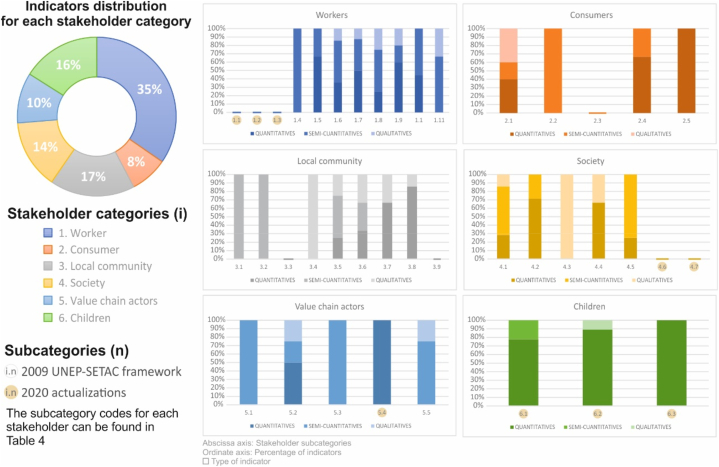
Analysis of indicators according to stakeholder categories and type of formulation.

These frameworks were compared with the reference standard of UNEP-SETAC and, in this way, the indicators proposed by each article within the reference framework were classified. As previous comparative analyzes on S-LCA frameworks, highlight the work carried out by Fürtner et al. [68]. A total of 172 different indicators were analyzed prioritizing those applicable to the product life cycle. The comparative results are shown in Table 4, according to the total or partial coverage of the reference framework, the equity of study of the stakeholders or interested groups (workers, local community society, consumers, value chain actors and children), the status of the positive indicators and, within the quantitative indicators -considered the most objective and reliable-which ones provide the most information -whether they are developed and are not case-specific or general indicators. In addition, the quantitative, qualitative or semi-quantitative nature of indicators was identified. This is represented in Fig. 9.

**Table 4:** Comparison between different reference frameworks and the one proposed by UNEP.

	UNEP-SETAC Life Cycle Initiative Framework	ODS framework	SEEbalance	PSIA	Social welfare-based framework

**1. Worker**	^(^^1.1)^Freedom of association and collective bargaining	•	•	•	
	^(^^1.2)^Child labour	•		•	•
	^(^^1.3)^Forced labour	•		•	•
	^(^^1.4)^Fair salary	•	•	•	
	^(^^1.5)^Working hours	•		•	
	^(^^1.6)^Equal opportunities/discrimination	•		•	•
	^(^^1.7)^Health and Safety	•		•	
	^(^^1.8)^Social benefits/social security	•			
	^(^^1.9)^Employment relationships	•			
	^(^^1.10)^Sexual harassment	•			
	^(^^1.11)^Smallholders including farmers			•	
**2. Consumer**	^(^^2.1)^Health and Safety	•	•	•	
	^(^^2.2)^Feedback Mechanism	•			
	^(^^2.3)^Consumer privacy	•		•	
	^(^^2.4)^Transparency	•			
	^(^^2.5)^End-of-life responsibility	•			
**3. Local community**	^(^^3.1)^Access to material resources	•		•	
	^(^^3.2)^Access to immaterial resources	•			
	^(^^3.3)^Delocalization and Migration	•			
	^(^^3.4)^Cultural Heritage	•			
	^(^^3.5)^Respect of indigenous rights	•			
	^(^^3.6)^Secure living conditions	•	•		
	^(^^3.7)^Safe and healthy living conditions	•		•	
	^(^^3.8)^Community involvement	•		•	
	^(^^3.9)^Local Employment	•	•	•	
**4. Society**	^(^^4.1)^Public commitments to sustainability issues	•			
	^(^^4.2)^Contribution to economic development	•			
	^(^^4.3)^Prevention and mitigation of armed conflicts	•			
	^(^^4.4)^Technological development	•			
	^(^^4.5)^Corruption	•			
	^(^^4.6)^Ethical treatment of animals	•			
	^(^^4.7)^Poverty alleviation	•			
**5. Value chain actors**	^(^^5.1)^Fair competition	•			
	^(^^5.2)^Promoting social responsibility	•			
	^(^^5.3)^Respect for Intellectual Property Rights	•			
	^(^^5.4)^Supplier relationships	•			
	^(^^5.5)^Wealth Distribution	•			
**6.Child.**	^(^^6.1)^Education provided in the local community	•	•		
				

^(6.2)^Health issues for children as consumers.

^(6.3)^Children's concerns about marketing practices.

#### 3.5.1 UNEP-SETAC Life Cycle Initiative Framework

UNEP-SETAC Life Cycle Initiative Framework is the most comprehensive and complete procedure in the current literature. It is disseminated in its three main documents: (1) Guidelines for Social Life Cycle Assessment of Products [12], (2) The Methodological Sheets for Subcategories in Social Life Cycle Assessment (S-LCA) [39] and (3) Guidelines for Social Life Cycle Assessment of Products and Organizations 2020 [13]. This framework is based on the concept of social sustainability as the "identification and management of impacts, both positive and negative, on people, represented as stakeholders". Therefore, it is a stakeholder-focused methodology, considering elements on which inclusion or exclusion in the scope of application must be justified [85].

The UNEP-SETAC life cycle initiative proposes a list of impact categories according to stakeholders or interest groups. These are further divided into specific impact sub-categories (human rights, working conditions, health and safety, cultural heritage, governance, socio-economic repercussions). Measurable impact indicators are utilized for each sub-category. For instance, under the subcategory "Workers," metrics include *Freedom of Association and Collective Bargaining, Child Labour, Fair Salary, Hours of Work, Forced Labour, Equal Opportunities/Discrimination, Health and Safety, and Social Benefit/Social Security*. These impact sub-categories are generally based on international agreements, such as the Universal Declaration of Human Rights or various ILO or OECD reports-to provide a solid foundation [12,85].

The first edition of the S-LCA guidelines proposes an indicator framework encompassing five stakeholder groups (workers, consumers, local community, society and value chain actors) and thirty-one impact sub-categories [12,39]. However, in the edition published in 2020, the interest group of “Children”, and nine additional subcategories (wealth distribution, Ethical treatment of animals, Poverty alleviation, among others), are considered, resulting in a framework of six stakeholder groups and forty impact subcategories as shown in Table 4.

#### 3.5.2 Integration of S-LCA with sustainable development goals

The SDGs, consisting of 17 internationally agreed goals with a target date of 2030, are characterized by social indicators, similar to the S-LCA [1]. Consequently, several studies have analyzed the implication of life cycle impacts on the satisfaction of the SDGs [8,90]. In 2018, this relationship was studied with respect to the S-LCA in order to cover all three aspects of sustainability. The study revealed that only 25 % of the SDGs are aligned with end-point impacts, while most of the sub-targets relate to system outputs in relation to policy plans and regulations. Considering this, a comprehensive framework was proposed to classify the targets in terms of mid-point and end-point impacts. Within this classification, it was concluded that the issue of forced labour is not adequately covered by the SDGs. Moreover, some of the SDGs are not addressed in the S-LCA, such as targets 2 and 11, and the indicators for targets 5, 9, 13 and 15.

The comparative assessment conducted in this review (see Table 4) reveals the scope of social indicators available in S-LCA concerni ng the overall SDGs. The results suggest that the S-LCA indicators do not adequately cover discrimination issues, unlike the SDGs, which measure discrimination across various aspects such as gender, indigenous communities, access to education, and income. S-LCA indicators related to SDGs 2, 13, 14, 15 and 17 -13, 14 and 15 were not found. These findings have been compiled in the guidelines as 'Positive indicators of type C' [13]. Furthermore, in the UNEP-SETAC guidelines update, the S-LCA subcategories are classified with respect to the 2030 Agenda framework, and all S-LCA subcategories are shown to be associated with each other, resulting in a different outcome compared to the studies discussed above. Although limited, several works attempting to address this gap by analyzing the relationship between S-LCA and the SDGs [53].

#### 3.5.3 Social welfare framework

Van Haaster, Ciroth, Fontes, Wood, and Ramírez [87] provide a systematic analysis of the general considerations of S-LCA. They are based on the concept of social well-being, which, according to the World Health Organization (WHO) in 2002, "is one of the 3 pillars that determine human health with a focus on quality of life. It includes education, equality, freedom of expression, poverty, slavery, and terrorism (OHCHR)". The results are excluded from biophysical aspects in human health by toxicity; this is justified by the possible overlap with the environmental scope of the LCA.

According to the WHO, the main categories of social well-being are as follow: 1) autonomy; 2) safety, security and tranquillity; 3) equality; and 4) participation and influence. Table 5 summarizes the relationship between the S-LCA guidelines indicators and categories 1 and 3. Category 4 was excluded because participation and influence are more related to stakeholders and not with the impact subcategories; however, their consideration could be useful in defining the objectives of the S-LCA as well as in the interpretation phase. On the other hand, category 2 is conceptually generic, and its classification within a specific group of the UNEP-SETAC framework is not possible.

**Table 5:** Extraction of indicators useful for S-LCA.

References	Principal aspects
[49,91]	They provide a developing country perspective, where a large part of the product life cycle is usually located.
[92] [68]	These are comprehensive and up-to-date indicator frameworks. Moreover, they focus on the production of bio-based products. These belong to the small percentage of case studies relating to products and not to services or other areas.
[93]	The life cycle of each of the main components of an ASUS computer is broken down and the S-LCA is carried out qualitatively for each of them at their place of production. Although qualitative, it is very complete and helps to identify possible indicators.
[94]	Selection of positive impact indicators found so far.
[56]	A framework focused on children as a group of interest is developed. This is The Sustainable Child Development Index (SCDI), established in previous studies by the same authors, applied to the context of an S-LCA.
[87]	Indicators based on a different framework than the UNEP-SETAC guidelines, starting from the concept of social welfare.
[14]	Study the indicators most used in the literature as well as other useful data analysis.

#### 3.5.4 Private sector frameworks

In addition to the three frameworks described in sections 3.5.1, 3.5.2 and 3.5.3, there are two frameworks developed with private initiative: (i) SEEbalance© method for Socio-Economic Efficiency Analysis in 2004 [88] and (ii) Product Social Impact Assessment (PSIA) of Pré Sustainability Assessment.

SEEbalance© method for Socio-Economic Efficiency Analysis in 2004 [88] was developed by BASF in cooperation with the Universities of Karlsruhe and Jena as well as the Öko-Institut e.V. This method reinterprets the product-related concepts of eco-efficiency and socio-efficiency by relating the ecological and social impacts occurring throughout the product life cycle to the end-customer's costs of purchasing, using, maintaining, and finally disposing of or reselling the product, 'total costs of ownership' [95]. Specifically, the analysis is developed from the results of an S-LCA and a social hotspot analysis, which are compared with the SDGs to obtain results, identifying gaps and discussing potentials for improvement.

Product Social Impact Assessment (PSIA) or Pré Sustainability Assessment was developed by PRé Sustainability, together with the Product Social Metrics Roundtable, which included multinationals such as Ahold, BASF or BMW Group. In 2013, they jointly published the Product Social Impact Assessment Handbook [80] in response to the lack of global standards for the assessment of social impacts at the product level. It is based on several of the principles proposed in the GRI guidelines (principles of balance, materiality, reliability, among others). The manual also includes 12 key principles which companies can socially assess their products.

Fürtner et al. [68] compare these two frameworks and the UNEP-SETAC guidelines [12,39]. The results show that the stakeholder “society” and “value chain actors” does not include, although the new group 'small-scale entrepreneurs' is created. On the other hand, the SEEbalance © method adds 'future generations' and 'international community' while excluding the group 'society'. Both the category 'small-scale entrepreneurs' and 'future generations' coincide with the updates proposed by the 'Guidelines for Social Life Cycle Assessment of Products and Organizations' 2020, analyzed in this research (see Table 4).

#### 3.5.5 Analysis of individual indicators proposed in scientific literature

An individual analysis was conducted on the indicators from the frameworks examined in this review, as listed in Table 5. The results are shown in Fig. 9. The classification was based on the stakeholder groups defined in the UNEP-SETAC framework, considering the indicators’ quantitative, semi-quantitative, and qualitative attributes [96]. This visual classification provides insights into the ongoing research efforts aimed at developing S-LCA. The figure comprises, firstly, a pie chart displaying the distribution of indicators available for each stakeholder group in S-LCA: workers, consumers, local community, society, value chain actors, and children. Secondly, the figure includes a detailed analysis of the indicators available for each stakeholder group, categorized according to their quantitative, semi-quantitative, or qualitative nature (indicated by high, medium, or low color intensity, respectively). Each subcategory was systematically enumerated and abbreviated as per Table 5. Furthermore, the six diagrams distinguish between the categories of the UNEP-SETAC framework from 2009 and recent updates in 2020. This representation offers a comprehensive overview of the diversity and accessibility of analysis indicators within each category, highlighting specific areas that require further attention and future development in the field of S-LCA.

The existing indicators are diverse and comprehensive enough for the implementation of an S-LCA. The worker interest group has the most complete set of indicators, while indicators for value chain actors and children need improvement in the medium term. Addressing the new challenges proposed in the UNEP-SETAC 2020 Guidelines, such as developing indicators for the stakeholder group 'children' or including positive social impacts, is necessary.

A gap is identified in the scientific literature regarding the consumer group, as well as the conception of the full social life cycle of industrial products, which is currently underdeveloped. It should be noted that the lack of availability of indicators in some stakeholders makes it challenging to calculate the quantitative social impact in the product's life cycle. In such situations, a qualitative or semi-quantitative assessment is applied. However, this procedure increases the complexity in aggregating results. Integrating both quantitative and qualitative indicators makes it difficult to find a proper balance between the two, leading to imprecise or non-approachable interpretation of results with a single social impact score [6].

### 3.6 Digital resources and development tools for S-LCA

Currently there are different calculation tools for S-LCA. These can be classified according to the following categories.

• S-LCA software: They are based on the software for the calculation of environmental impacts in the life cycle. These tools allow you to simplify and automate the S-LCA methodology. Besides, they present the results in a graphical form for easy interpretation. Each software uses its own database and indicator framework; some of them allow you to incorporate third-party databases.
• Databases: Online storage media for data at local, national, and international, or sector level. They extract the information necessary (inputs and outputs) for the calculation of impact indicators in a specific area and/or sector.
• Reference documents that support decision-making on the use of categories, sub-categories and indicators. They are usually international and/or national or sectoral regulations; or reports and/or statements from various international organizations.
• Life Cycle Organizations focus on the research, dissemination, and real-world application of S-LCA. In particular, there are two important partnerships: UNEP Life Cycle Initiative and Social Value Initiative, organised by Pré Sustainability, which integrates different companies.

The situation of each group is reviewed below, setting out the main conclusions according to their quality and availability.

#### 3.6.1 S-LCA software

The most complete software is highlighted in this review in relation to the scope of impact calculation and the exhaustiveness required in a social life cycle methodology. The three currently most appropriate software for S-LCA are OpenLCA developed by GreenDelta, SimaPro from PRé Consultants and Gabi from ThinkStep. SimaPro enables S-LCA to be carried out in all three dimensions (environmental, social, and economic), determine KPIs (Key Performance Indicator) and perform sustainability reports and generate Environmental Product Declarations (EDP). It supports ecoSpold and csv data formats and is compatible with the social database PSILCA and SHDB. Gabi is focused on environmental LCA, but also covers the social aspects related to the stakeholder group 'workers' through the Life Cycle Working Environment (LCWE) methodology. Open LCA is an open source and the most complete in terms of social databases. Although less well known, other examples of life-cycle-based sustainability analysis software are SEEbalance from BASF and Pro-suite from PRé Sustainability. Both use slightly different Life Cycle approaches from what is proposed in the UNEP-SETAC Guidelines. Table 6 summarize a comparative analysis with the positive and negative aspects of these professional tools [[97], [98], [99], [100], [101]].

**Table 6:** Comparative of ACVS software.

	openLCA	SimaPro	Gabi
**Company**	Germany, GreenDelta	Netherlands, PRé Consultants	Germany, ThinkStep
**Brief description**	LCA (environmental, social and economic), carbon and water footprint analysis. Development of economic models, among other functions.	Determination of KPI and perform LCA (environmental, social and economic), sustainability reports and generate Environmental Product Declarations (EDP).	LCA (environmental, economic, social) and Life Cycle Working Environment (LCWE). Calculate Carbon Footprints, Water Footprints, Water Footprints and Environmental Footprints. Preparation of life cycle reports (circularity, ecodesing, etc.)
**Firt version**	2006	1990	1989
**Database format**	ILCD, ecoSpold v1, v2, csv, Excel, JSONLD. Sirve para PSILCA y SHDB.	ecoSpold, csv. Sirve para PSILCA y SHDB.	ILCD, EPD, ecoSpold v1, GPR, gbx.
**Result model**	Process modeling and diagrams for each unit process	Matrices for modeling unit processes	Process modeling and diagrams for each unit process
**Result presentation**	Sankey diagrams and bar graphs. Tables for LCI	Sankey diagrams and bar graphs. Tables for LCI	Sankey diagrams and bar graphs. Tables, and automatic flow balances for LCI
**Social data set**	Social hotspots database (SHDB); PSILCA	Social hotspots database (SHDB); PSILCA	LCWE data
**Sensitivity of results**	Scenario analysis, Monte Carlo test, Pedigree Matrix, uncertainty distribution test.	Scenario analysis, Monte Carlo test, Pedigree Matrix	Scenario analysis, Monte Carlo test. Percentage deviations can be used for inventory flows
**User support**	Automatic calculation once the LCI has been modeled. and Downloadable databases, online technical support, tutorials, FAQ and forum.	Automatic calculation once the LCI has been modeled. Downloadable databases, online technical support, tutorials, FAQ and forum.	Automatic calculation once the LCI has been modeled. Downloadable databases, online technical support, tutorials, FAQ and forum.
**Cost**	Open license	Private license	Private license
**Advantages**	Fast network calculation for processes Wide variety of databases, both free and private access. Datasets can be easily imported and exported. Possibility to share datasets online Possibility of generating EPD	Adapted to ISO 14067, ISO 14040, Possibility of generating EPD. Good documentation of data sets Integrated with Ecoinvent database. Most datasets are unit processes.	Easily import and export datasets. Good documentation of data sets. Professional database with hundreds. of data sets and many extension databases. Possibility of generating EPD. It is possible to acquire personalized databases.
**Disadvantages**	Some datasets are not open Access. Some datasets are poorly documented. Normalization and weighting factors are not available for the ILCD/PEF method.	High investment cost Limited number of dataset formats Professional software, requires specialization and prior training.	High investment cost. Most of the data sets are aggregated. Professional software, requires specialization and prior training.

#### 3.6.2 S-LCA databases

Currently, there are a wide variety of social databases at the global level. This review focus on those specific to S-LCA, i.e. those designed according to the UNEP-SETAC guidelines. These are the PSILCA and the Social Hotspots Database (SHDB). According to Huarachi et al. [37] both marked the beginning of the S-LCA (the period from 2013 to 2016) where the methodology started its path towards standardisation. In addition, there are many social databases. These include, for example, RepRisk, Maplecroft, Ecovadis, Wage indicator, Country profiles, Eora, Fairwage, Sedex, Soca, Organisation for Economic Co-operation and Development (OECD) database, International Labour Organisation (ILO) database, United Nations data, World Bank Group (WBG), Human Rights Watch and Amnesty International data.

Social Hotspot Database (SHDB) [77] was created by the consultancy firm New Earth to provide generic data to S-LCA practitioners to identify social hotspots. The database currently consists of 20 aspects and approximately 100 indicators, selected through the criterion of availability. It covers a total of 140 countries and regions and 57 economic sectors. Each dataset contains information on its quality, which can be calculated for the entire system-product (in addition to information on sources, collection time, and the basis for risk assessment).

Product Social Impact Life Cycle Assessment (PSILCA) database was developed by the consultancy GreenDelta [102]. It provides statistical data for 88 indicators in 25 sub-categories based on the UNEP-SETAC guidelines. Cover a total of 14.838 sectors for 189 countries, including very detailed sectors. Each data set shows the source, the specific year, and data point, and an overall assessment scheme. As a complement, 'GreenDelta' developed an interactive map to show the amount of information managed by countries for each database. However, it only shows Nexus databases, where the vast majority is environmental LCA data [103].

It is important to emphasize that the LCI (life cycle inventory) is not made exclusively with databases. The 2020 guidelines by UNEP-SETAC describes the "S-LCA data ecosystem" [13], which distinguishes between data sources and tools to obtain data. These tools may involve interviews, surveys, audit results, scientific publications, etc. The selection of these tools should be based on S-LCA's scope and the available resources. Additionally, in a social impact study, triangularisation is commonly employed. This involves combining results from various data collection methods to enhance the validity of the research.

#### 3.6.3 Reference documents

When conducting an S-LCA, it is advisable to refer complementary resources, such as guides, documents on conventions, reports, regulations at international, national, and sectoral level. Considering the universal rights perspective, ILO Conventions on labor rights or standpoint of companies (such as Corporate Social Responsibility), the reference documents can be classified as follows:,.

- **Universal consensus on social issues**: These are normative instruments, representing agreement between subjects of international law with the aim of establishing rights and obligations among the participating parties. In this review, the conventions developed by the United Nations Organization are considered, which includes representatives from all over the world, 193 states. Therefore, these conventions are regarded as a solid fundamental basis for the development of social indicators. There are summarized in Table 7.

**Table 7:** International conventions on social issues.

ILO Conventions on Workers' Rights	Freedom of association and the effective recognition of the right to collective bargaining	Freedom of Association and Protection of the Right to Organise Convention (No. 87)
		Right to Organise and Collective Bargaining Convention (No. 98)
	Abolition of forced labour	Forced Labour Convention (No. 29)
		Abolition of the Forced Labour Convention (No. 105)
	Equality	Discrimination (Employment and Occupation) Convention (No. 111)
		Equal Remuneration Convention (No. 100)
	Eradication of Child Labour	Convention on the minimum age (No. 138)
		Worst Forms of Child Labour Convention (No. 182)
Universal Chart of Human Rights	Universal Declaration of Human Rights [104]	
	International Covenant on Civil and Political Rights [105]	
	International Covenant on Economic, Social, and Cultural Rights (ICESCR) [106]	
Transforming our World: The 2030 Agenda for Sustainable Development [1]		
Tripartite Declaration of Principles concerning Multinational Enterprises and Social Policy [107]		
Guiding Principles on Business and Human Rights. Implementing the United Nations “Protect, Respect and Remedy” Framework [108]

**Table 8:** Standards, programs, and guidelines on Corporate Social Responsibility.

Standards, programmes, and guidelines	Description
G3, G3.1, or G4 Guidelines of the Global Reporting Initiative (GRI)	Guidelines for the preparation of annual sustainability reports applicable to all types of organizations [86]
ISO 26000	It sets out guidelines for applying social responsibility to all types of enterprise. Main areas: organisational governance, human rights, labour practices, the environment, fair operating practices, consumer issues, community involvement, and development [85]
Global Social Compliance Programme (GSCP) *Belonging to the Consumer Goods Forum*	It works on the problems arising from audits: fatigue, quality, points for improvement, etc. It promotes the standardisation of methods and tools such as *supplier* self-assessment [85]
SA 8000 *Belonging to the organization 'Social Accountability International' (SAI)*	An independent certification standard that promotes the development, implementation, and maintenance of socially responsible workplace practices. It provides guidelines for ensuring fair compensation, preventing workplace discrimination, etc. [92,116].
OECD Guidelines for Multinational Enterprises. *The importance of responsible business conduct.* They are mentioned in the *articles* [53,116,117]	
World Business Council for Sustainable Development (WBCSD) reports to guide the development of socio-economic and social capital impact assessments, e.g, “Measuring socio-economic impact. A Guide for Business from 2013”.	
International Finance Corporation (IFC) Performance Standards on Environmental and Social Sustainability

- **International reports and congresses on social issues:** Updated reports collected for the social indicators:
  o Sustainable Development Goals Report [109].
  o International Labour Conference [110].
  o United Nations General Assembly [109].
  o Human Development Report
  o Child Labour. Global Estimates 2020, trends and the road forward [111].
  o World Development Report [112].
  o State of European Technology [113].
- **Global Policies Based on Life-Cycle Thinking**:
  o The 10 Year Framework of Programmes on Sustainable Consumption and Production (10YFP) based on the Rio+20 World Summit and launched in the Marrakech Process: (1) Guidance for the providing product sustainability information [114]; and (2) Sustainable Public Procurement Programme [115].
- **Specific business context, CSR:** Corporate Social Responsibility (CSR) is a relatively contemporary concept. Introduced in organizations as a new business philosophy based on respect, ethical values, environment care, and other aspects of sustainability. The S-LCA can be considered a CSR tool. Table 8 summarizes the most representative reference documents.

### 3.7 Case studies

**Fig. 10:**
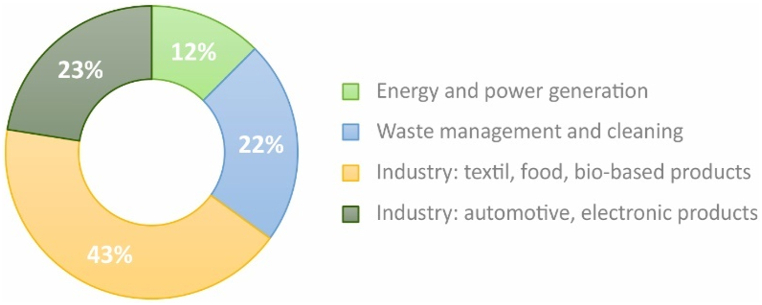
Research effort dedicated to the application of S-LCA.

Finally, this review examines a collection of articles that apply S-LCA in various sectors. These case studies utilize the foundational principles, methodologies, and indicators discussed in earlier sections to measure social impact. Table 9 provides an overview of the forty-one selected cases; and in order to assess the research effort dedicated to the application of S-LCA in case studies, the different fields were ranked based on the number of publications, resulting in the distribution shown in Fig. 10. Only a limited number of publications of this nature have been identified, concluding that the application of S-LCA to industrial products is still an emerging area of research. According to the sample, the bio-based product sector has the highest number of cases, confirming the finding expressed in the study of Huarachi et al. [37] which highlights the bioeconomy as a new trend in S-LCA studies.

**Table 9:** Classification of the case study sample by industry sector.

Industry Sector	References
Textile industry	[13,53,118,119]
Food industry	[17,18,20,[120], [121], [122], [123]]
Electronic products	[93,[124], [125], [126]]
Waste management	[45,49,[126], [127], [128], [129]]
Automotive	[22,119,[130], [131], [132]]
Energy generation	[52,54,56,133,134]
Chemical industry	[[135], [136], [137], [138]]
Bio-based products and economy	[15,16,68,89,[139], [140], [141]]

It is important to highlight that there is a limited number of publications that specifically address a S-LCA of industrial products. This observation is evident both in the broader literature review, where the field of 'design practices and management ' is particularly underrepresented (Fig. 4), and in the specific sample of reviewed articles. The case studies within the sample primarily focus on waste management and bio-based products. S-LCA has demonstrated its worth in sustainable development process. As evidenced by the case studies analyzed, S-LCA facilitated more informed and ethical decision-making during the design and manufacturing stages. In several cases, S-LCA revealed areas necessitating improvements in labor practices, working conditions, and relationships with local communities. This detailed information is useful for adjusting product life cycle management strategies and improving the organization's corporate social responsibility initiatives. Furthermore, S-LCA promoted innovation by identifying opportunities to optimize processes and reduce negative social impacts. It can be concluded that, in the case studies analyzed, S-LCA served as a guide for improving socially responsible practices in the industrial sectors examined.

## 4 Discussion

Social Life Cycle Assessment indeed originates from Environmental Life Cycle Assessment, sharing the same ISO 14040 framework. For the adaptation of the procedure, the 'Guidelines for Social Life Cycle Assessment' provide guidance and key aspects of conducting a full S-LCA. Furthermore, the 2020 update of the framework includes advancements and new paradigms that have emerged as the methodology has matured, expanding the methodological approach to an organizational level known as SOLCA [12,13]. On the other hand, the methodological sheets provide support for the application of S-LCA, particularly in specific contexts. They offer proposed indicators, data classification, generic and specific data collection sources, as well as additional documents for further elaboration. Possible extensions are also considered, including characterization and aggregation models. Additionally, in December 2021, an updated version of the methodological sheets was published by the same life-cycle initiative [39]. However, there is currently no standardisation for S-LCA in ISO standards. The development of harmonized procedure for S-LCA is an ongoing area of research within the field of sustainability. On the other hand, there is currently no standardized direct relationship between S-LCA and the Sustainable Development Goals (SDGs). While there is a hierarchical structure that includes various levels of social indicators, as well as general SDGs and SDG indicators, it is important to note that the SDG framework covers all three dimensions of sustainability. However, it is commonly acknowledged in studies that the Life Cycle Sustainability Assessment (LCSA) approach is more suitable for integrating the three dimensions of sustainability compared to S-LCA alone.

Due to the flexible relative nature of the framework proposed by the 'Life Cycle Initiative' and the lack of standardization of the S-LCA procedure, it is common for each case study to develop its own specific version of S-LCA. This reflects the authors' requirement to adapt the methodology to various contexts and research objectives. The situation emphasizes the importance of optimizing the current procedure. Additionally, the lack of standardization poses a challenge when comparing results obtained from different S-LCA models and when seeking to obtain comprehensive results. Based on this, several authors theorize the process of developing a standardization framework and engage in debates regarding the possibility of establishing a harmonized S-LCA approach. The aim is to enable its flexible implementation across different industrial sectors without the need to create custom method variants for each specific case.

In relation to the calculation of social impact through the indicators included in the framework, and as shown in Table 5, the stakeholder categories that are more extensively developed are the groups Workers and Local Community, with 42 % and 29 % of available indicators respectively. Meanwhile, the indicators for the group Society cover 15 % of the categories, while Consumers and Value Chain Actors are the least studied, with 7 % and 6 % respectively. These results agree with the conclusions of Fürtner et al. [68] and they align with the sampling in Table 9, where the categories of Consumers, Value Chain Actors, and Society are consecutively considered to a lesser extent. It is important to note that the availability of indicators is directly related to the quality of the social impact results. Similarly, the impact categories that have received the least attention in terms of the number of indicators are Consumers and Value chain actors, in contrast to the emphasis placed on the Workers group. The indicators proposed by Chang et al. for the category Children [56], while valuable for other sectors, are found to be generic and not well-suited for the context of industrial products. Some impact categories are not adequately covered in the calculation, particularly in relation to the new updates of the S-LCA guide. For instance, subcategories like 'Labour relations' and 'sexual harassment' for the Workers group require further development. Additionally, there is a need for improvement other subcategories, such as Working conditions and Regional value creation, as identified by Fürtner et al. and Siebert et al. [68,92]. This gap in the scientific literature regarding Consumers and Value chain actors, as well as the lack of studies focusing on the stage of product use or the relationship between value chain actors, has been previously addressed in previous research studies [14,117].

Additionally, several studies have emphasized the lack of inclusion of positive indicators in existing frameworks (e.g. job creation, efforts of the sector in environmentally friendly technological development, gender equity or quality of life), despite being a characteristic of S-LCA [142]. Since the initial approach introduced by Norris and Siebert [85,92], there have been very few studies on this topic [37,143]. The updated guidelines incorporate the theoretical basis that has been developed thus far, although they do not specifically address its application within the methodological framework [144]. Di Cesare conducts a comprehensive literature review of the positive indicators employed to date [94]. The review concludes that the majority of positive indicators fall under the category of Value chain actors due to their capacity to measure company behavior, which is not regulated by specific standards. However, positive indicators are often qualitative or have not been well-developed [93,94].

The analysis of the digital resources available in the market to carry out an S-LCA showed that the most comprehensive software tools that closely adhere to the UNEP guidelines S-LCA development are 'OpenLCA' and 'SimaPro'. Both analyze environmental and economic LCA, and can be used for other purposes such as calculating carbon footprint or developing environmental product declarations. OpenLCA stands out for its versatility and ease of access as it is open-source software compatible with various data formats. SimaPro is one of the pioneering LCA software tools and was the first to incorporate S-LCA. Gabi is another widely used software for conducting environmental S-LCA. It includes the option to assess social impacts specifically related to the stakeholder group of workers through the Life Cycle Working Environment (LCWE) method, although it does not provide a complete S-LCA functionality. Regarding databases, there are two main databases that provide data for S-LCA: SHDB and PSILCA. They utilize other generic databases, either at the international or sector level. PSILCA covers a broader range of territories and economic sectors compared to SHDB, which focused on more specific sectors. In terms of the number of indicators they collect, both databases have approximately 100 indicators following the UNEP framework. However, it is important to note that data collection for S-LCAs should not rely exclusively on databases. It is necessary to combine other more direct tools and triangulating methods to ensure robust results (UNEP & SETAC, 2020).

Below, considering the importance of establishing a calculation based on quantitative indicators, the obtained results are discussed, highlighting the future challenges for each stakeholder.

- **Workers:** There is a lack of quantitative indicators for *Freedom of association and collective bargaining* and *Social benefits.* The new subcategories introduced in the 2020 model *Sexual harassment*, *Labour relations*, and *Smallholders including farmers* have not been adequately addressed. It is noteworthy that the “right to strike”, as outlined in the International Covenant on Economic, Social, and Cultural Rights, is not mentioned in the subcategory Freedom of association and collective bargaining [105]. Likewise, further exploration of the indicator "dangerousness of work" is needed within the category of "child slavery". In terms of 'fair wages', the PSILCA database distinguishes between the 'minimum wage required by law', 'prevailing industrial wage', and 'living wage', with the recommendation to prioritize the latter [102]. The impact of overtime teleworking, which has become more prevalent since the 2020 pandemic [110], should also be addressed. For 'forced labour', PSILCA proposes a generic index on 'proximity to trafficking in persons' based on efforts to meet the minimum standards set by The Trafficking Victims Protection Act (TVPA). In relation to 'equal opportunities', it is suggested to include discrimination based on political beliefs or religion. Lastly, additional impact subcategories are proposed, such as 'fixed recruitment rate' and 'level of job satisfaction', which encompass indicators not classified in the UNEP-SETAC framework, under the 'working conditions' category.
- **Consumers:** Only quantitative indicators have been defined for the subcategory of 'health and safety'. In terms of 'feedback mechanisms', two qualitative indicators of positive impacts are identified, and proposals include the use of satisfaction surveys. Regarding the category of 'transparency', it is suggested to utilize the S-LCA as a social sustainability report. For 'end-of-life accountability', the focus is on the presence of a feedback system, the publication of an end-of-life management manual, and incorporating end-of-life design considerations. However, no indicators related to 'consumer privacy' were found, indicating the need for the development of indicators in this category.
- **Society:** There are no defined quantitative indicators for 'Prevention and mitigation of armed conflicts'. Additionally, there are no proposed metrics for the new subcategories introduced in 2020, namely 'Ethical treatment of animals' and 'Poverty reduction' Regarding the category of 'public commitment to sustainability issues', most of the indicators are semi-quantitative measures of positive impact, with a particular emphasis on quantifying the quality and implementation of codes of conduct. In terms of measuring 'contribution to economic development', indicators focus on support to MSMEs (Micro, Small, and Medium Enterprises) and employment generation. Fürtner et al. propose the category of “Contribution to oppression” inspired by the 'CIRI project' [68]. Finally, for the category *Prevention and mitigation of armed conflict*, indicators are being sought that assess the probability of product control of control by criminal organizations (mafias). For the category *Corruption*, it is suggested to measure the potential risk using indicators provided by the NGO 'Transparency International'.
- **Local community:** There are no defined quantitative indicators for *Access to material and immaterial resources* and *Cultural heritage*, and no proposals have been made for the subcategories of *Relocation and migration* and *Safe living conditions*. For *Access to intangible resources* and/or *Cultural heritage*, it is suggested to research on indicators related to local festivities and traditions. Regarding 'healthy and safe living conditions', it would be beneficial to explore changes in food prices (food security) and measure the reduction in the use of hazardous substances. Furthermore, it is interesting to set an indicator for the rate of employment generation in the local community within the subcategory of *Safe and secure living conditions* based on the CIRI [68].
- **Value chain actors:** Quantitative indicators are only available for the subcategory *Promotion of social responsibility*, specifically on the number and percentage of audited suppliers. Within the sub-category *Wealth distribution*, it is feasible to assess the impact of introducing the product into the global economy by examining the difference in GDP between developed and developing countries. The subcategory *Fair competition* relies on semi-cuantitatives indicators and currently lacks well-developed quantitative measures. It primarily focuses on policies aimed at preventing anti-competitive behaviour. There is a notable absence of studies the measurement of local intellectual property, compensation policies and practices, and the existence of interaction with suppliers.
- **Children:** All generic quantitative indicators (such as measuring children's enrolment rates by country for the subcategory Education provided in the local community) can be compared with rates for children of company workers [49,91]. However, there is a lack of specific indicators for the subcategory of *Problems for children's health as consumers.* Similarly, indicators for the subcategory of *Children's concerns about marketing practices* tend to be general indicators that affect children as stakeholders, including economic status and environmental aspects.

The analysis of the digital resources available in the market to carry out an S-LCA showed that the most comprehensive software tools that closely adhere to the UNEP guidelines S-LCA development are 'OpenLCA' and 'SimaPro'. Both analyze environmental and economic LCA, and can be used for other purposes such as calculating carbon footprint or developing environmental product declarations. OpenLCA stands out for its versatility and ease of access as it is open-source software compatible with various data formats. SimaPro is one of the pioneering LCA software tools and was the first to incorporate S-LCA. Gabi is another widely used software for conducting environmental S-LCA. It includes the option to assess social impacts specifically related to the stakeholder group of workers through the Life Cycle Working Environment (LCWE) method, although it does not provide a complete S-LCA functionality. Regarding databases, there are two main databases that provide data for S-LCA: SHDB and PSILCA. They utilize other generic databases, either at the international or sector level. PSILCA covers a broader range of territories and economic sectors compared to SHDB, which focused on more specific sectors. In terms of the number of indicators they collect, both databases have approximately 100 indicators following the UNEP framework. However, it is important to note that data collection for S-LCAs should not rely exclusively on databases. It is necessary to combine other more direct tools and triangulating methods to ensure robust results (UNEP & SETAC, 2020).

Based on the review results, it is evident that there are currently no specialized S-LCA methodology for assessing industrial products. Two main areas of future research are proposed.

1. Define a comprehensive set of quantitative and representative indicators specifically tailored for assessing the life cycle of industrial products. Taking into account the identified shortcomings, the following sub-areas of research are identified:
- Propose specific indicators to complement those new categories suggested in the 2020 guidelines, particularly for the stakeholder group Children and the additional sub-categories less development. In this regard, it is advisable to consider the indicators proposed by PSIA and SEEbalance in the categories 'aspects', 'small-scale entrepreneurs' and 'future generations' respectively.
- Develop indicators that specifically target the often overlooked stakeholders: Value chain actors, Consumers and Society.
- In the 'consumers' domain, it is necessary to introduce indicators from the perspective of user interaction during the use phase of the life cycle, such as usability, inclusive design, and design for special populations. Additionally, there is a need to explore the inclusion of positive impact indicators, which have not been widely applied yet, based on the UNEP-SETAC 2020 guidelines.
- Study new and/or existing impact pathways to derive robust indicators for PSS-LCA. Specifically, it is suggested to explore the manual calculation of the DALY (Disability Adjusted Life Year) approach or the 'fair wage' indicator proposed by Neugebauer et al. [145] as potential indicators.
2. Develop a socio-economic impact calculation tool tailored to the life cycle of a product based on the procedure outlined in the updated UNEP guidelines. The existing tools are complex and primarily focused on organizational management level.

## 5 Conclusions

This work presents a comprehensive review of the current state of S-LCA in the context of industrial products. Currently, the application of S-LCA methodology to the life cycle of products yields results with high uncertainty, especially in comparative analyses, limiting its use to individual assessments and hindering the comparison and selection of alternatives considering the social impact generated.

This complexity arises from the lack of standardization in the procedure and the absence of an unify group of quantitative social indicators. Given this situation, it is necessary to develop a framework specifically tailored for industrial products. This framework should incorporate the updated guidelines provided by UNEP-SETAC in 2020 and address the various challenges identified by the scientific community in developing robust quantitative indicators.

Additionally, it is necessary to define the role of positive impact indicators within S-LCA and address the ongoing discussion regarding the inclusion or exclusion of commonly used subcategories, as identified in the methodological guidelines, or explore alternative options within the product lifecycle. Furthermore, it is important to develop a set of indicators for less developed stakeholders and, particularly, focus on formulating realistic and robust quantitative indicators for each area of the framework, ensuring their applicability to any industrial product context. This review provides a compilation of resources intended to serve as a guide towards achieving the required standardization.

The objective use of Life Cycle Assessment (LCA), in its comprehensive form, as a tool for calculating and achieving the Sustainable Development Goals (SDGs) by 2030, demands urgent standardization. Furthermore, it is crucial to acknowledge the significant role of the design stage in minimizing the social impact throughout the product's life cycle. In addition to the scientific progress related to the standardisation and reduction of uncertainty of S-LCA results, it is necessary to develop new methodologies that include principles, strategies, or guidelines applicable to the product design phase, with the aim of optimizing solutions from a social point of view for all stakeholders in the life cycle of the system (society, community, workers, suppliers, and consumers).

## Data availability statement

Data included in article/supp. material/referenced in article.

## Funding

This work was supported by the VII-PPITUS of the 10.13039/100009042Universidad de Sevilla, grant number: 2022/00000329. Responsible Researcher: Peralta, E (mperalta1@us.es).

## CRediT authorship contribution statement

**Carmen Mármol:** Formal analysis, Data curation. **Amanda Martín-Mariscal:** Writing – review & editing, Writing – original draft, Methodology, Investigation, Conceptualization. **Alberto Picardo:** Writing – review & editing, Software, Resources, Methodology, Formal analysis, Data curation. **Estela Peralta:** Writing – review & editing, Supervision, Methodology, Investigation, Funding acquisition, Formal analysis, Data curation, Conceptualization.

## Declaration of competing interest

The authors declare that they have no known competing financial interests or personal relationships that could have appeared to influence the work reported in this paper.
